# Low dynamic muscle strength and its associations with fatigue, functional performance, and quality of life in premenopausal patients with systemic lupus erythematosus and low disease activity: a case–control study

**DOI:** 10.1186/1471-2474-14-263

**Published:** 2013-09-08

**Authors:** Sandor Balsamo, Licia Maria Henrique da Mota, Jozélio Freire de Carvalho, Dahan da Cunha Nascimento, Ramires Alsamir Tibana, Frederico Santos de Santana, Ricardo Lima Moreno, Bruno Gualano, Leopoldo dos Santos-Neto

**Affiliations:** 1 Graduate Program in Medical Sciences, School of Medicine, Universidade de Brasília (UnB), Brasília, Brazil; 2 Rheumatology Service, Hospital Universitário de Brasília (HUB) UnB, Brasília, Brazil; 3 Physical Education Department, Centro Universitário UNIEURO, Brasília, Brazil; 4 Rheumatology Division, Clínica de Oncologia (CLION), Salvador, Brazil; 5 Graduate Program on Physical Education, Catholic University of Brasilia, Brasilia, Brazil; 6 School of Physical Education, UnB, Brasília, Brasilia, Brazil; 7 School of Physical Education and Sports, University of Sao Paulo (USP), Sao Paulo, Brazil; 8 School of Medicine, Rheumatology Division, USP, Sao Paulo, Brazil

**Keywords:** Dynamic muscle strength, Fatigue, Quality of life, Systemic lupus erythematosus, Functional performance

## Abstract

**Background:**

The purpose of the present study was to compare dynamic muscle strength, functional performance, fatigue, and quality of life in premenopausal systemic lupus erythematosus (SLE) patients with low disease activity *versus* matched-healthy controls and to determine the association of dynamic muscle strength with fatigue, functional performance, and quality of life in SLE patients.

**Methods:**

We evaluated premenopausal (18–45 years) SLE patients with low disease activity (Systemic lupus erythematosus disease activity index [SLEDAI]: mean 1.5 ± 1.2). The control (n = 25) and patient (n = 25) groups were matched by age, physical characteristics, and the level of physical activities in daily life (International Physical Activity Questionnaire IPAQ). Both groups had not participated in regular exercise programs for at least six months prior to the study. Dynamic muscle strength was assessed by one-repetition maximum (1-RM) tests. Functional performance was assessed by the Timed Up and Go (TUG), in 30-s test a chair stand and arm curl using a 2-kg dumbbell and balance test, handgrip strength and a sit-and-reach flexibility test. Quality of life (SF-36) and fatigue were also measured.

**Results:**

The SLE patients showed significantly lower dynamic muscle strength in all exercises (leg press 25.63%, leg extension 11.19%, leg curl 15.71%, chest press 18.33%, lat pulldown 13.56%, 1-RM total load 18.12%, *P* < 0.001-0.02) compared to the controls. The SLE patients also had lower functional performance, greater fatigue and poorer quality of life. In addition, fatigue, SF-36 and functional performance accounted for 52% of the variance in dynamic muscle strength in the SLE patients.

**Conclusions:**

Premenopausal SLE patients with low disease activity showed lower dynamic muscle strength, along with increased fatigue, reduced functional performance, and poorer quality of life when compared to matched controls.

## Background

It has been speculated that fatigue, a symptom frequently observed in approximately 80% of SLE patients [1], may contribute to a reduction in physical fitness (i.e., muscle weakness and low cardiovascular capacity), which, in turn, leads to an impairment in the performance of activities of daily living and consequently, in the overall quality of life [2].

Most studies examining the relationship between physical fitness and overall health in SLE patients have only addressed cardiovascular fitness [2]. However, decrements in muscle strength have also been strongly associated with a greater number of cardiovascular events [3,4] and early mortality [5,6] in several populations. Currently, the association between dynamic muscle strength, fatigue, functional performance, and quality of life in SLE patients remains unknown.

Previous studies have demonstrated that SLE patients have decreased isometric muscle strength when compared to healthy controls [7,8]. These studies evaluated muscle strength through isometric tests. Dynamic strength tests may be more informative than static tests for physical function evaluation because daily living functioning primarily encompasses dynamic rather than isometric contractions [7,8]. These previous studies also did not control for important confounding factors that affect physical performance, such as obesity [3], fibromyalgia [9,10], perimenopause [11], smoking [12], using beta-blockers [13] and statins [14], activities of daily living and physical activity level [15], and socioeconomic status [7]. In the present study, we did correct for these confounding factors; therefore, their influence on the dependent variables may be considered minimal.

Thus, the objective of this study was twofold: 1) to compare dynamic muscle strength, functional performance, fatigue, and quality of life in premenopausal SLE patients with low disease activity *versus* matched-healthy controls and 2) to determine the association between dynamic muscle strength and fatigue, functional performance, and quality of life in these patients. We hypothesised that premenopausal SLE patients withp low disease activity would present reduced dynamic muscle strength when compared with their healthy peers. Furthermore, we hypothesised that low dynamic muscle strength would be associated with fatigue, poor functional performance, and impaired quality of life in SLE patients.

## Methods

### Study design and patients

The study was conducted between January 2009 and January 2011. A single examiner analysed the medical records and conducted structured interviews with 240 patients who were being followed in the outpatient Clinic of Rheumatology at the Brasilia University Hospital (HUB, Brasilia/Brazil). The interviews included socioeconomic status (e.g., education, employment, and income). Disease activity was assessed by the Systemic Lupus Erythematosus Disease Activity Index (SLEDAI) [16]. The control group was recruited through email, leaflets, and posters. Healthy women were primarily matched by physical activity levels, age and physical characteristics (i.e., body weight and body fat). The participants provided signed informed consent. The study was approved by the local ethics committee and was in accordance with the Helsinki Declaration of 1975, as revised in 1983.

### Inclusion criteria

All participants were premenopausal women. The SLE patients met the American College of Rheumatology (ACR) criteria [16], with stable disease (i.e., no flare-ups or changes in medication for at least 3 months before entering the study) [17]. Additionally, all participants had not engaged in regular exercise programs for at least six months prior to the study [10].

### Exclusion criteria

The following exclusion criteria were applied: SLEDAI > 5 (n = 19), serum creatinine ≥ 265 mmol/l, myositis, haematocrit ≤ 30%, nephritis and/or leukopenia (n = 13), history of myocardial infarction, cardiomyopathy, hypertension and/or the use of beta-blockers (n = 18), type 2 diabetes mellitus (n = 6), neurological diseases (n = 4), hypothyroidism (n = 5), fibromyalgia (n = 23), osteoporosis (n = 6), rheumatoid arthritis (n = 3), Sjögren’s syndrome (n = 2), cancer (n = 1), age < 18 years (n = 2) and > 45 years (n = 39), residence located far (other state) from the research centre (n = 40), body mass index (BMI) < 18 kg/m^2^ (n = 1) and > 30 kg/m^2^ (n = 8), smoking (n = 10), pregnancy (n = 1), and engaged in regular exercise (n =14).

### Methods

The participants who met the inclusion criteria visited the laboratory on three different occasions in 48 to 72-hour intervals at the same time (2–4 pm). Two days prior to the tests, the participants were recommended to avoid intensive exercise, caffeine or alcohol intake. The evaluations were not performed during the menstrual period. On the first day, fatigue symptoms, quality of life, and physical activity level were assessed [15,18,19]. Additionally, anthropometric measurements and functional performance tests were performed. The patients were also familiarised with the one-repetition maximum strength tests (1-RM). On the second and third days, the 1-RM test and re-test were performed, respectively.

### Anthropometric measurements

A single examiner evaluated height, weight, and BMI. Body fat was estimated using the skinfold measurement, as previously described [20].

### Fatigue symptoms, quality of life, and physical activity level

All questionnaires were administered prior to the physical tests. The following fatigue scales were used: Fatigue Severity Scale (FSS) [1], which consists of a 9-question questionnaire with a score ranging from 1 to 7. Quality of life was assessed using the Short-Form Health Survey 36 (SF-36) [19], which consists of 36 items grouped into eight sub-domains (i.e., physical functioning, role-physical functioning, bodily pain, general health, vitality, social functioning, role-emotional functioning, and mental health). The SF-36 score ranges from 0 to 100, with a higher score indicating better health-related quality of life. The level of physical activities of daily living was evaluated using the short version of the International Physical Activity Questionnaire (s-IPAQ) [18], which consists of questions regarding the frequency (i.e., days per week) and duration (i.e., minutes per day) of occupational and recreational activities of daily living and structured exercise programs according to the physical activity level. The subjects were classified into three categories: active, irregularly active, and inactive.

### Functional performance tests

Physical function was assessed through the following battery of tests. *The* 30-s chair stand test evaluated the number of times that a subject was able to stand from a standard chair and sit down again in 30 seconds [21]. The *30-s arm curl test* assessed upper-body muscle function by the number of arm curl repetitions performed with 2-kg dumbbell for 30 seconds [21]. The *handgrip strength test* (Takei Kiki Kogyo Co., Ltd., Japan) evaluated the maximal isometric strength of the dominant hand using a calibrated dynamometer. The volunteers stand erect holding the dynamometer parallel to the side, with the dial facing away from the body. Each participant performed the test twice, with a 1-minute rest period between the measurements. The best value was chosen for the analysis. The grip position of the TKK dynamometer was adjusted to the individual’s hand size [22]. The Timed Up and Go (TUG) *test* assessed the time that a subject required to rise from a standard arm chair, walk 3 meters away, turn, return, and sit down again [23]. The *one foot balance test with eyes closed* assessed balance by having subject stand on one foot with eyes closed for up to 30 seconds [24]. The *sit and reach test* evaluated flexibility using the modified chair sit-and-reach test, as previously described [25].

### Dynamic muscle strength

The 1-RM test was used to determine the dynamic muscle strength of the upper- and lower-limbs [26] using conventional weight machines (Johnson Health Technologies, Taiwan) [5,27-29]. Prior to the 1-RM test, two light warm-up sets were performed in two-minute intervals. Then, the participants were given up to five attempts to achieve the 1-RM load (i.e., the maximum weight that could be lifted once using proper technique), with a five-minute interval between the attempts. The 1-RM tests were conducted for leg press, chest press, leg extension, lat pulldown, and leg curl exercises. The strength tests were performed on two different days and were separated by a 48–72 minute period, which allowed the calculation of the test-retest reliability (i.e., intra-class coefficient [ICC]) for both groups [28].

Sample size calculation was cited in a previous study [30] and assumed an effect size of 0.97 between groups; thus, a minimum sample of 25 volunteers for each group was required to provide 90% power (5% significance). The non-paired Student's T-test or Mann–Whitney U-test was used to compare the groups. Physical activity levels and socioeconomic status data were analysed by Pearson's chi-squared test.

The forward stepwise linear regression model was used to investigate the relationship between muscle strength (as the dependent variable) *versus* functional performance (i.e., 30-s chair stand test and handgrip tests), fatigue (i.e., FSS score), and quality of life (physical functioning subscale from SF-36) for the SLE patients. The regression model was applied as the 1-RM-total (muscle strength: sum of total load lifted in the 1-RM tests [i.e., leg press + leg extension + leg curl + chest press + lat pulldown]), according to Ruiz et al. [5]. Normally distributed data are expressed as the mean±SD, and non-normally distributed data are expressed as median and interquartile range. The significance level was set at 5%. The analyses were performed using the software SAS for Windows 9.2 (SAS Institute Inc., Cary, NC, USA).

## Results

### Patients

Twenty-five premenopausal SLE patients with a low disease activity (SLEDAI = 1.5 ± 1.2, range = 0–5, 9 of 25 patients) and a disease duration of 5.3±4.6 years (range = 1–20 years) participated in this study. The patients were taking corticosteroids (21/25 [84%], dose = 6.07 ±2.1 mg/day, range = 5–20 mg/day), azathioprine (8 of 25 patients [32%], dose = 87.50 ±46.8 mg/day, range 50–200 mg/day), chloroquine diphosphate (17/25 [68%], dose = 205.88 ± 66.4 mg/day), and hydroxychloroquine (2 of 25 patients [8%], dose = 400 ±0.0 mg/day). The primary characteristics of the SLE patients and the matched healthy controls are shown in Table 1.

**Table 1:** **Main characteristics of SLE patients *versus *matched-healthy controls**

**Variable**	**SLE**	**CONTROLS**	**Difference between means**	***P*-value**
	**(n = 25)**	**(n = 25)**	**(95% CI)**^**#**^	
Age, years, median (IQR)†	29.9 (6.8)	29.2 (8.1)		0.7671
Body mass, kg	57.7 ± 6.7	58.3 ± 8.2	0.69 (-3.6, 4.9)	0.7462
Height, cm	158.1 ± 0.1	158.3 ± 0.9	-0.01 (-0.1, 0.1)	0.6573
Lean body mass, kg	38.1 ± 4.8	38.5 ± 3.8	0.5 (-2.1, 2.9)	0.6966
BMI, kg/height^2^	23.1 ± 2.9	23.5 ± 3.3	0.5 (-13, 2.2)	0.5998
Body fat, %	33.5 ± 9.2	33.2 ± 8.6	-0.3 (-5.4, 4.7)	0.8997
Sum of skinfolds, mm	79.8 ± 19.4	79.6 ± 18.4	-0.1 (-10.9, 10.6)	0.9769
Thigh skinfold, mm	23.1 ± 5.6	22.3 ± 6.4	-0.8 (-4.2, 2.6)	0.6351
Right thigh circumference, cm	55.2 ± 4.2	56.2 ± 3.6	1.1 (-1.2, 3.3)	0.3404
Systolic blood pressure, mmHg	108.0 ± 10.2	106 ± 8.6	-2.3 (-7.7, 3.1)	0.3818
Diastolic blood pressure, mmHg, median (IQR)†	68.0 (11.4)	69.0 (9.8)		0.7410
Heart rate, bpm	80.0 ± 10.3	81.0 ± 14.9	1.2 (-6.1, 8.5)	0.7432

* Values are expressed as the mean ± SD unless otherwise stated. † These variables are not normally distributed and therefore, are expressed as median. # Calculated only when Student’s t-test was used. *SLE* systemic lupus erythematosus, *CI* confidence interval, *IQR* interquartile range, *BMI* body mass index, *mmHg* millimetres of Mercury, *BPM* beats per minute.

### Socioeconomic status

The SLE patients had lower educational level than the controls, *P* < 0.05). The groups did not differ regarding other socioeconomic status variables (*P* > 0.05) (Table 2).

**Table 2:** Socioeconomic characteristics of the SLE patients and healthy controls

**Variable**	**Group**				
	**SLE**		**CONTROLS**		***P*-value**
	**N**	**%**	**N**	**%**	
Educational level					
Literacy, 0 to 4 years	3	12	0	0	0.0003
Primary, 5 to 8 years	8	32	0	0	
Secondary, 9 to 12 years	9	36	23	92	
University, 12 years >	5	20	2	8	
Employment					
Paid	14	56	19	76	0.0702
Unemployed	11	44	6	24	
Income†					
No income	10	40	6	24	0.5345
Up to 1 minimum salary	6	24	8	32	
From 1 to 2 minimum salaries	6	24	8	32	
Over 2 minimum salaries	3	12	3	12	

† Brazilian minimum salary equivalent to US$280.00 in 2009 (R$ 465.00).

### s-IPAQ

None of the participants had engaged in regular exercise programs for at least six months prior to the study. The level of physical activities of daily living was similar between the groups (*P* = 0.12). In the SLE group, 17 of 25 (68%) patients were active, 3 of 25 (12%) were irregularly active, and 5 of 25 (20%) were inactive. In the control group, 23 of 25 (92%) subjects were active, 1 of 25 (4%) was irregularly active, and 1 of 25 (4%) was inactive.

### Fatigue symptom and quality of life

When compared with the controls, the SLE patients had significantly higher FSS score (*P* < 0.01). The SLE patients also had poorer quality of life parameters when compared with the controls, (all *P* < 0.05), except for vitality and bodily pain domains (both *P* > 0.05) (Table 3).

**Table 3:** Functional capacity, fatigue scores and quality of life in the SLE patients and controls*

**Variable**	**SLE**	**CONTROLS**	**Difference between means**	***P*-value**
	**(n = 25)**	**(n = 25)**	**(95% CI)**^**#**^	
Functional performance				
Handgrip strength, kg	24.2 ± 4.9	27.1 ± 4.7	2.8 (0.1; 5.5)	0.0464
30-s chair stand test, repetitions	19.6 ± 5.6	24.1 ± 3.7	4.5(1.7; 7.2)	0.0018
30-s arm curl test, repetitions	20.5 ± 3.3	24.6 ± 3.6	4.1 (2.1; 6.1)	0.0001
Timed Up and Go, s	5.3 ± 0.4	5.0 ± 0.6	-0.3 (-0.6; -0.0)	0.0495
Flexibility sit and reach, cm	24.0 ± 9.5	29.0 ± 9.4	5.0 (-0.40; 10.4)	0.0680
30-s balance, s	17.6 (9.8)	17.0 (9.1)	-	0.7848
Fatigue symptom				
FSS, median (IQR)†	3.5 (1.2)	2.5 (0.9)	-	0.0043
Quality of life, SF-36				
Physical functioning, median (IQR)†	61.6 (24.4)	81.2 (14.5)	-	0.0029
Role-physical functioning, median (IQR)†	53.0 (41.03)	78.0 (25.3)	-	0.0375
Bodily pain, median (IQR)†	64.4 (25.7)	72.9 (22.0)	-	0.2752
General health	51.1 ± 17.8	67.4 ± 16.3	16.3 (6.6; 26.0)	0.0014
Vitality, median (IQR)†	54.8 (11.5)	55.2 (10.3)	-	0.9686
Social functioning, median (IQR)†	68.4 (24.0)	83.8 (18.3)	-	0.0266
Role-emotional functioning	41.1 (39.9)	73.2 (36.1)	-	0.0073
Mental health, median (IQR)†	50.0 (13.2)	58.5 (10.6)	8.5 (1.7; 15.3)	0.0150

* Values expressed as the mean ± SD, unless otherwise stated. † These variables are not normally distributed and therefore are expressed as median. # Calculated only when Student’s t-test was used. *SLE* systemic lupus erythematosus, *CI* confidence interval, *FSS* fatigue severity scale, *IQR* interquartile range.

### Functional performance

When compared with the controls, the SLE patients had a significantly lower functional performance in general (*handgrip test* = -10.35%, TUG test = - 5.94%, *30-s chair timed-stand test* = - 18.60%, *30-s arm curl test* = - 16.58%, all *P* < 0.05), except for balance and flexibility (both *P* > 0.05) (Table 3).

### Muscle strength (1-RM)

The ICC for the 1-RM test was 0.98 (CI 0.60, 0.99) and 0.99 (CI 0.86, 0.98) for the SLE and control groups, respectively. The SLE patients had a significantly lower 1-RM than the controls in all exercises (1-RM = -25.63%, leg extension = -11.19%, leg curl = -15.71%, chest press = -18.33%, lat pulldown = -13.56%, 1-RM [total load] = - 18.12%, 1-RM/relative [total load/body weight = - 17%, all *P* < 0.05]) (Table 4).

**Table 4:** Dynamic muscle strength (1-RM) in SLE patients and controls*

**Variable (1-RM)**	**SLE**	**CONTROLS**	**Difference between means**	***P*-value**
	**(n = 25)**	**(n = 25)**	**(95% CI)**^**#**^	
Leg press, kg	71.1 ± 18.6	95.6 ± 19.7	24.5 (13.6; 35.5)	< 0.0001
Leg extension, kg	64.7 ± 10.6	72.9 ± 13.9	8.1 (1.1; 15.2)	0.0242
Leg curl, kg	30.2 ± 5.2	35.8 ± 6.9	5.6 (2.1; 9.1)	0.0022
Chest press, kg	34.7 ± 7.2	43.1 ± 7.9	8.4 (4.1; 12.7)	0.0002
Lat pulldown, kg	36.2 ± 6.4	41.8 ± 5.8	5.6 (2.2; 9.1)	0.0019
1-RM-total, kg	47.4 ± 8.1	57.8 ± 8.7	10.4 (5.7; 15.0)	< 0.0001
1-RM-total, kg/kg of body weight	0.8 ± 0.1	1.0 ± 0.1	0.2 (0.1; 0.2)	< 0.0001

* These values are expressed as the mean SD unless otherwise stated. † These variables are not normally distributed and therefore, are expressed as median. # Calculated only when Student’s t-test was used. SLE = systemic lupus erythematosus; CI = confidence interval of 95%; 1-RM = 1-RM consists of performing a full range of motion with the greatest possible load; 1-RM -total = sum of total load lifted in the 1-RM tests (i.e., leg press, leg extension, leg curl, chest press, and lat pulldown); 1-RM-total, kg/kg of body weight = median of the sum of the load of the 1-RM tests in leg press, leg extension, leg curl, chest press, and lat pulldown divided by body weight.

### Linear regression model

The final model for dynamic muscle strength, which included the functional performance tests (handgrip and timed chair stands), SF-36 (physical role functioning and emotional role functioning scores), and FSS scores, accounted for 52% of the variance in dynamic muscle strength (F= 5.62, P = 0.02, VIF < 10, Table 5). Unexpectedly, physical role functioning was inversely related to 1-RM in the final model. However, physical role functioning seemed to be a weak independent factor in 1-RM change (R^2^ = 0.14; = -0.38), therefore the role of this variable as a predictor in the final model must be interpreted with caution. The errors in the model were independently distributed (Durbin-Watson = 2.19), and multicollinearity was not detected (VIF = 1.0).

**Table 5:** Association between dynamic muscle strength and functional performance tests, SF-36 subscale and fatigue score

**Dependent variable**	**Independent variable**	$\hat{\beta }$	**Standard error**	**R**^**2**^	***P*-value**
1-RM-total, kg	Role-physical functioning (SF-36)	-0.38	0.09	0.14	< 0.001
	Handgrip test	2.09	0.61	0.22	0.0027
	Role-emotional functioning (SF-36)	0.23	0.08	0.34	0.0125
	Timed chair-stand test	1.61	0.53	0.42	0.0065
	Fatigue (FSS)	-9.80	4.13	0.52	0.0218

$\hat{\beta }$ **=** parameter estimate, R^2^ = coefficient of determination; 1-RM = one repetition maximum is the largest possible load of a particular movement; 1-RM -total = sum of total load lifted in the 1-RM tests (i.e., leg press, leg extension, leg curl, chest press, and lat pulldown); *SF*-*36* self-administered health questionnaire - Short Form Health Survey 36, *FSS* fatigue severity scale.

### Adverse events

No adverse events were recorded during the experimental period. Additionally, none of the patients reported joint pain at time of testing.

## Discussion

The novel finding of this study is that premenopausal SLE patients with low disease activity show lower dynamic muscle strength (upper- and lower-limb) when compared with their healthy peers. Furthermore, we provided the evidence that lower dynamic muscle strength was associated with fatigue, low functional performance, and poor quality of life (namely, role-emotional functioning) in SLE patients.

Our results are in agreement with those by Tench et al. [7] and Stockton et al. [8], who demonstrated that SLE patients have lower isometric muscle strength when compared with healthy controls. However, the aforementioned studies evaluated muscle strength using isometric tests. In this regard, one may argue that dynamic strength tests may be more informative than static tests in terms of physical function evaluation because daily living functioning primarily encompasses dynamic rather than isometric contractions [7]. In fact, the significant association between dynamic strength (i.e., 1-RM) and physical function assessments (i.e., chair timed-stands) observed in the current study further supports this notion.

Fatigue scores (as assessed by the FSS questionnaire) lower than 4.0 suggest that fatigue is not severe enough to limit participation in daily living physical activities. Conversely, FSS scores higher than 4.0 suggest that fatigue is perceived to adversely affect the ability to engage in physical and social activities [1]. In the current study, however, the SLE patients scored 3.5 on average (with 15 of 25 patients having FSS scores lower than 4). However, the patients experienced decreased physical function, low dynamic muscle strength capacity, and poor quality of life, suggesting that either “residual” fatigue or other factors (e.g., long-term medication or systemic inflammation) may have contributed to the poor health-related findings demonstrated in this study. Further studies must elucidate the role of fatigue on health-related parameters in SLE patients.

We observed that even with low fatigue and low disease activity scores, 20% (5 of 25) of the SLE patients showed handgrip strength between 17 and 20 kg. Notably, these values are considered a marker of sarcopenia [23]. The handgrip strength test has been considered a clinical marker of mobility [23,31] and lower limb muscle strength [23]. Moreover, a 5-kg increase in handgrip strength has been associated with a significantly reduced mortality risk [6]. This fact, along with the fact that the handgrip strength test has been proven reliable in SLE patients [32], make this simple and inexpensive tool an emerging marker of clinical relevance. Further prospective studies should test its ability as a prognostic marker in SLE.

Our study must be interpreted in light of its strengths and limitations. Although a few studies have also demonstrated lower physical function in SLE patients [7,8], these studies did not control for important confounding factors that affect physical performance, such as obesity [3], fibromyalgia [9,10], perimenopause [11], smoking [12], use of beta-blocker [13] and statins [14], activities of daily living, physical activity level [15], and socioeconomic-status [7]. In the present study, we did correct for these confounding factors; therefore, their influence on the muscle strength may be considered minimal.

However, this study is not without limitations. First, the cross-sectional nature of this study precluded us to establish cause-effect relationships between muscle strength and health-related parameters in SLE patients (e.g., role-emotional functioning from SF-36). Second, our homogeneous sample comprised premenopausal SLE patients with low disease activity and who were free of comorbidities and associated diseases. Therefore, one cannot extrapolate the present results to older or younger patients with more severe disease. Finally, our sample size was relatively low. Further studies should test the accuracy of our multivariate model in a larger patient cohort.

## Conclusions

In conclusion, the current study provided novel evidence that lower- and upper-body dynamic muscle strength is reduced in premenopausal SLE patients with low disease activity when compared with their controls. Importantly, we also demonstrated that lower dynamic muscle strength is associated with fatigue, low functional performance, and poor quality of life in SLE patients.

## Competing interests

This research received no specific grant from any funding agency in the public, commercial, or not-for-profit sectors. The authors declare no competing interest.

## Author’s contributions

SB, LSN, DCN, RAT and FSS were responsible for concept and design, statistical expertise, data analysis and interpretation, helped write the manuscript. SB and LSN were responsible for data analysis and interpretation and helped write the manuscript, BC, JFC, RCM and LMHM were significant manuscript reviewers/ revisers and were responsible for data analysis and interpretation. SB, LSN and BC were significant manuscript reviewers/revisers and were responsible for data acquisition. BC, JFC, RCM and LMHM were a significant manuscript reviewer/reviser and were responsible for data acquisition, analysis and interpretation. All authors have read and approved the manuscript for publication.

## Pre-publication history

The pre-publication history for this paper can be accessed here:

http://www.biomedcentral.com/1471-2474/14/263/prepub
